# High Dose of Lamivudine and Resistance in Patients with Chronic Hepatitis B

**DOI:** 10.1155/2014/615621

**Published:** 2014-09-30

**Authors:** Hamid Ullah Wani, Saad Al Kaabi, Manik Sharma, Rajvir Singh, Anil John, Moutaz Derbala, Muneera J. Al-Mohannadi

**Affiliations:** Department of Medicine, Division of Gastroenterology, Hamad Medical Corporation (HMC), 2 South 2, P.O. Box 3050, Doha, Qatar

## Abstract

*Background.* Lamivudine is the most affordable drug used for chronic hepatitis B and has a high safety profile. With the daily dose of 100 mg there is progressive appearance of resistance to lamivudine therapy. In our study we used 150 mg of lamivudine daily as a standard dose which warrants further exploration for the efficacy of the drug. *Aims of the Study.* To assess the efficacy of lamivudine 150 mg daily on resistance in patients with chronic hepatitis B. *Methods.* This retrospective study consists of 53 patients with chronic hepatitis B treated with 150 mg of lamivudine daily. The biochemical and virological response to the treatment were recorded at a 1-year and 2-, 3-, 4-, and 5-year period and time of emergence of resistance to the treatment was noted. *Results.* The mean age of the patients was 54 years with 80% being males. The resistance to lamivudine 150 mg daily at 1 year and 2, 3, and 5 years was 12.5%, 22.5%, 37.5%, and 60%, respectively, which is much less compared to the standard dose of 100 mg of lamivudine. *Conclusions.* Lamivudine is safe and a higher dose of 150 mg daily delays the resistance in patients with chronic hepatitis B.

## 1. Introduction

Chronic hepatitis B remains the most common serious health problem in the world, especially in the Asia Pacific region. Worldwide, there are 350 million people with chronic carrier of HBV. Treatment of HBV is relatively safe and easy compared to hepatitis C treatment, but the drug resistance is the main problem. The lamivudine and telbivudine are prone to develop resistance rapidly. Since the introduction of lamivudine, treatment of chronic hepatitis B has been characterized by a rapid increase in the number of available antiviral drugs, all belonging to the class of HBV polymerase inhibitors. Due to better tolerance and more convenient administration compared to interferon, HBV polymerase inhibitors today account for the vast majority of prescribed therapies for chronic hepatitis B in Western countries [1, 2]. However, long-term suppression of HBV is needed, particularly in HBeAg-negative patients harboring the precore mutant.

Lamivudine (LAM) was the first approved HBV polymerase inhibitor. It is characterized by good clinical tolerability, moderate antiviral efficacy, and rather quick development of resistance. Approximately 20% of patients treated with LAM develop resistance to LAM by 1 year and 70% to 80% by 5 years after the start of treatment [3].

Preliminary data indicate that the development of multiple lamivudine associated mutations may even reduce the efficacy of tenofovir therapy [4]. However, we have a good number of patients who are on lamivudine therapy with excellent viral response who need a continuous followup to observe the development of LAM resistance.

The clinical endpoints of chronic hepatitis B treatment are still not well defined [5, 6]. In HBeAg-positive patients, HBeAg seroconversion has been shown to be associated with a reduction in liver-associated morbidity and increased survival [7]. Thus, HBeAg seroconversion is considered a clinical endpoint in this group of patient population and discontinuation of HBV polymerase inhibitors is recommended 6–12 months after HBeAg seroconversion in patients who have not developed liver cirrhosis [8–10]. Treatment endpoints in HBeAg-negative hepatitis B in most cases are restricted to sustained normalization of ALT levels and suppression of HBV DNA, as HBsAg seroconversion is rare with current treatment options [3, 7]. Consequently, treatment duration and endpoints are more difficult to define in these patients. Most guidelines therefore recommend indefinite treatment of patients with HBeAg-negative chronic hepatitis B.

## 2. Materials and Methods

This retrospective study included 53 patients with chronic hepatitis B who were on lamivudine treatment since June 2005 at the Department of Gastroenterology, Hamad Medical Corporation. Before the start of lamivudine treatment all patients were HBsAg positive, had detectable levels of HBV DNA level >5 to 10log⁡ copies/mL, and had elevated liver enzymes about 3 to 5 times the upper limit of normal. All patients received lamivudine in a single daily dose of 150 mg.

The patients with the following conditions were excluded from the study:

1. coinfection with hepatitis C, hepatitis D, and human immunodeficiency virus,
2. association with other forms of liver diseases, such as alcoholic liver disease, drug-induced hepatitis, or autoimmune hepatitis,
3. previous treatment of HBV with interferon and nucleos(t)ide analogs other than LAM.

The patients who did not have a regular followup on the medical records and the patients who had no clinical and laboratory assessments at regular intervals were excluded.

All of these patients had a followup after every 3 to 6 months with routine biochemical liver function tests and serum HBV DNA levels. Serum HBV DNA levels were quantified at baseline and at each follow-up visit using the COBAS Ampli Prep or COBAS Taqman HBV test (Roche Molecular System) [11, 12].

The (i) biochemical response (normalization of serum alanine aminotransferase (ALT) level), (ii) complete virological response (undetectable serum HBV DNA by real-time polymerase chain reaction, <100 copies/mL), and (iii) virological breakthrough were recorded in all patients. Out of 50 patients, 40 patients had a regular five-year followup available in the medical records.

Virological breakthrough was defined as an increase in serum HBV DNA of more than 1log⁡ copies/mL from the nadir of the initial response. A flare-up of hepatitis was defined as an increase in ALT level to more than 3 times the upper limit of normal.

In this study we do not have molecular studies available for lamivudine resistance. The time of “virological breakthrough” and “flare-up” of hepatitis was taken as resistance to lamivudine.

The study protocol was reviewed and approved by the Institutional Review Board of Hamad Medical Corporation.

## 3. Statistical Methods

Descriptive statistics in the form of mean, standard deviations, and range are calculated for interval variables, whereas counts and percentages are performed for categorical variables. Kaplan Meier curve has been performed to see probability of not having resistance to lamivudine at different points of months. SPSS 20.0 Statistical Package is used for the analysis.

## 4. Results

The study population consists of 53 patients with chronic hepatitis B who were on lamivudine since June 2005. Out of 53 patients, 40 patients fulfilled the inclusion criteria. The mean age was 54 years with 80% being males. Fourteen patients (35%) were from the state of Qatar and 26 patients (65%) were expatriates. Twenty-five patients (62.5%) were HBeAg + and 15 patients (37.5%) were HBeAg − (Table 1). Before starting lamivudine treatment, all patients were having elevated liver enzyme ranging from 3 to 5 times the upper limit of normal and HBVDNA levels >2,000 IU/mL. Mean duration of lamivudine treatment was 60 months. None of the patients received interferon therapy or any other antiviral drug during this period. Seven patients had liver biopsy which was showing fibrosis stages 1 to 2 and inflammation grades 2 to 3 on Metavir Score system (Table 2).

All patients (Table 3) had biochemical normalization within 3 to 6 months of initiation of lamivudine therapy. Overall (87%) patients were having a virological and biochemical response at 12 months of lamivudine treatment and 2 patients were having early viral breakthrough at 6 months after a partial HBV-DNA suppression. The response to lamivudine treatment was 77.4% at 24 months, 62.5% at 36 months, 50% at 48 months, and 40% at 60 months (Table 3).

**Figure 1:**
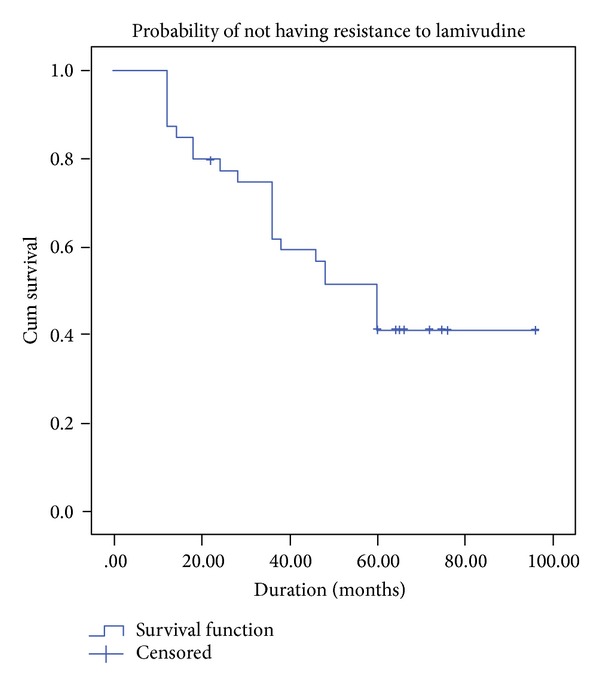
Kaplan-Meier curve showing probability of not having resistance to lamivudine.

There is progressive evolution of lamivudine resistance reaching up to 50% at 48 months and 60% at 60 months (Figure 1). Three patients achieved HBeAg seroconversion to anti-HBeAg, none had HBsAg seroconversion to antiHBs antibody positivity during these 5 years of treatment. Two patients died with multifocal hepatocellular carcinoma even after HBV-DNA suppression.

## 5. Discussion

Lamivudine was the first approved polymerase inhibitor for chronic hepatitis B. It is characterized by excellent tolerability with minimal or no side effects [2, 13]. There is rapid development of antiviral resistance to the standard dose of 100 mg of lamivudine in patients with chronic hepatitis B. Twenty (20%) patients developed resistance within one year of treatment which can progress to 70 to 80% at 4 to 5 years [6, 14]. The resistance rates have been higher in HBeAg positive patients [15, 16]. HBsAg loss and seroconversion to anti-HBsAg antibody may occur spontaneously in 1–3% of cases per year, usually after several years with persistently undetectable HBV DNA [3, 14].

In our study with the lamivudine dose of 150 mg daily the resistance at 1 year was only 12.5% compared to 20 to 24% with standard lamivudine dose. The resistance was also delayed at 2 and 3 years with the 150 mg lamivudine treatment and was 22.5% and 37.5% which is much less compared to the standard dose of 100 mg of lamivudine. The main result of our study is that patients who received high dose of lamivudine have lower rate of resistance (60%) over a mean duration of 60 months.

Torre et al. showed more profound suppression of viral replication with a lamivudine dose of 300 mg once daily [17]. Ha et al. showed that an initial high dose of 300 mg of lamivudine over a period of 2 weeks followed by 100 mg daily, compared to standard dose, has a lower rate of resistance (60% versus 76%) [18].

Adefovir, entecavir, and tenofovir are commonly used antivirals in patients with lamivudine resistance, although the development of the resistance is delayed and is less extent compared to lamivudine and telbivudine [19–21]. The lamivudine, even with dose of 150 mg, is safe in patients with end stage renal disease. Lamivudine mutations have been shown to confer cross-resistance to telbivudine, emtricitabine, and entecavir [22, 23].

The treatment endpoints of chronic hepatitis B are also not well defined. HBe antigen and hepatitis B surface antigen (HBsAg) seroconversion are markers of disease control and immunity. In our study, 7.5% achieved HBeAg seroconversion but none had HBsAg seroconversion during these 5 years of lamivudine treatment.

Lamivudine is the most cost-effective treatment for the chronic HBV [24, 25]. Although the new agents, like entecavir and tenofovir, appear more effective, they are more expensive than lamivudine. Selecting between these agents completely depends upon the available health care budget and willingness to pay. For poor patients, like in our study, who cannot afford the costly drugs, it appears more cost-effective to start with lamivudine than adefovir and entecavir. In one meta-analysis of cost-effective strategy, 3/6 studies that evaluated the lamivudine against other drugs find it as a dominant strategy [26]. As recommended by current management guidelines, lamivudine once daily is used for an indefinite period in patients with cirrhosis [27–29].

The important limitation of our study is that being a retrospective study there is no other available group for the comparison; however, the objective of the study was to see the effect of high dose of lamivudine on the resistant pattern of chronic hepatitis B.

## 6. Conclusions

Our study showed that 150 mg of lamivudine delayed the appearance of resistance in chronic hepatitis B. Lamivudine is very cheap compared to new antiviral drugs, has high safety profile, and has good compliance as compared to other new drugs. With this current available evidence, we consider that lamivudine as an antiviral treatment for chronic hepatitis B is a cost-effective intervention for many health care systems, including ours.

## Figures and Tables

**Table 1:** Demographic and clinical characteristics of the patients.

	Number	Range
Age	40	27–79 years Mean 54 years ± SD 8.94
Gender	40	Male 32 (80%) Female 8 (20%)
HBSAg	40	40 positive (100%)
HBSAb	40	32 positive (80%) 8 negative (20%)
HBeAg	40	25 positive (62.5%) 15 negative (37.5%)
HBeAb	40	15 positive (37.5%) 25 negative (62.5%)

**Table 2:** Biochemical and virological characteristics of the patient prior to treatment.

Disease duration	Years 5 (1–10) years

ALT IU/L	120 (80–150) IU/L
Duration of treatment	60 months
HBV DNA IU/mL	2.0 × 10^3–10^ IU/mL
Previous interferon therapy	0

**Table 3:** Treatment (biochemical and virological response).

	12 months	24 months	36 months	48 months	60 Months
HBV DNA IU/mL Below detection level	35/40 (87.5%)	31/40 (77.4%)	25/40 (62.5%)	20/40 (50%)	16/40 (40%)
Biochemical activity (high ALT)	2/40	normal	normal	normal	normal
HBsAg seroconversion	0%	0%	0%	0%	0%
HBeAg seroconversion	0%	0%	1	2	3
Viral breakthrough	5/40 (12.5%)	9/40 (22.5%)	15/40 (37.5%)	20/40 (50%)	24/40 (60%)
